# Both variants of *A1CF* and *BAZ1B* genes are associated with gout susceptibility: a replication study and meta-analysis in a Japanese population

**DOI:** 10.1007/s13577-021-00485-4

**Published:** 2021-01-31

**Authors:** Makoto Kawaguchi, Akiyoshi Nakayama, Yuka Aoyagi, Takahiro Nakamura, Seiko Shimizu, Yusuke Kawamura, Mikiya Takao, Takashi Tamura, Asahi Hishida, Mako Nagayoshi, Mitsuo Nagase, Keiko Ooyama, Hiroshi Ooyama, Nariyoshi Shinomiya, Hirotaka Matsuo

**Affiliations:** 1 Department of Integrative Physiology and Bio-Nano Medicine, National Defense Medical College, 3-2 Namiki, Tokorozawa, Saitama 359-8513 Japan; 2 Laboratory for Mathematics, Premedical Course, National Defense Medical College, Tokorozawa, Saitama Japan; 3 Department of Preventive Medicine, Nagoya University Graduate School of Medicine, Nagoya, Aichi Japan; 4 Nagase Clinic, Tokyo, Japan; 5 Ryougoku East Gate Clinic, Tokyo, Japan

**Keywords:** Gout/hyperuricemia, *BAZ1B/WSTF*, *MLXIPL/ChREBP*, Apolipoprotein B (ApoB), *ABCG2/BCRP*

## Abstract

Gout is a common type of acute arthritis that results from elevated serum uric acid (SUA) levels. Recent genome-wide association studies (GWASs) have revealed several novel single nucleotide polymorphism (SNPs) associated with SUA levels. Of these, rs10821905 of *A1CF* and rs1178977 of *BAZ1B* showed the greatest and the second greatest significant effect size for increasing SUA level in the Japanese population, but their association with gout is not clear. We examined their association with gout using 1411 clinically-defined Japanese gout patients and 1285 controls, and meta-analyzed our previous gout GWAS data to investigate any association with gout. Replication studies revealed both SNPs to be significantly associated with gout (*P* = 0.0366, odds ratio [OR] with 95% confidence interval [CI]: 1.30 [1.02–1.68] for rs10821905 of *A1CF*, *P* = 6.49 × 10^–3^, OR with 95% CI: 1.29 [1.07–1.55] for rs1178977 of *BAZ1B*). Meta-analysis also revealed a significant association with gout in both SNPs (*P*_*meta*_ = 3.16 × 10^–4^, OR with 95% CI: 1.39 [1.17–1.66] for rs10821905 of *A1CF*, *P*_*meta*_ = 7.28 × 10^–5^, OR with 95% CI 1.32 [1.15–1.51] for rs1178977 of *BAZ1B*). This study shows the first known association between SNPs of *A1CF*, *BAZ1B* and clinically-defined gout cases in Japanese. Our results also suggest a shared physiological/pathophysiological background between several populations, including Japanese, for both SUA increase and gout susceptibility. Our findings will not only assist the elucidation of the pathophysiology of gout and hyperuricemia, but also suggest new molecular targets.

**Supplementary Information:**

The online version contains supplementary material available at 10.1007/s13577-021-00485-4.

## Introduction

Gout is a common disease which displays severe non-infectious acute arthritis and results from elevated serum uric acid (SUA) level, or hyperuricemia [1]. Recent genetic studies including genome-wide association studies (GWASs) have revealed several genes to be associated with SUA levels [2–7] as well as clinically-defined gout [8–14]. Of these, Köttgen et al. [7] reported 18 novel loci associated with SUA in European, Indian, African-American and Japanese populations. Two of them, rs10821905 of *A1CF* (chromosome 10q11.23) and rs1178977 of *BAZ1B* (chromosome 7q.11.23), showed the statistically significantly greatest and the second greatest effect size for increasing SUA level in a Japanese population [7]. While they showed a nominally significant association with gout in individuals of European ancestry, their association was not clarified in Japanese gout cases.

With clinically defined Japanese gout cases, we have identified several genes through a candidate gene approach [15–19] which are associated with gout. Many urate transporter genes, such as *SLC22A12/URAT1* and *SLC2A9/GLUT9*, whose dysfunctional variants cause renal hypouricemia [20–22] are also reported to have an association with gout and hyperuricemia.

This prompted us to examine the association between common variants of *A1CF*, *BAZ1B* and gout using clinically-defined Japanese gout patients through a candidate gene approach, and to meta-analyze it with our previous gout GWAS data [10].

## Methods

### Patients and controls

1411 male Japanese patients in total were recruited from the outpatients of the gout clinics of Ryougoku East Gate Clinic (Tokyo, Japan), Nagase Clinic (Tokyo, Japan), Wakasa Clinic (Saitama, Japan) and Tokorozawa Central Hospital (Saitama, Japan). All the subjects had been diagnosed with primary gout according to the criteria established by the American College of Rheumatology [23]. As the control group, 1,285 Japanese males without a history of gout or hyperuricemia (SUA levels > 7.0 mg/dL) were selected from participants in the Nagoya and Shizuoka area in the Japan Multi-Institutional Collaborative Cohort Study (J-MICC Study) [24, 25]. The mean age and standard deviation of cases and controls were, respectively, 48.2 ± 11.7 and 53.4 ± 9.9 years, and their mean body-mass indexes were 25.2 ± 3.7 and 22.9 ± 2.9 kg/m^2^, respectively.

### Genetic and statistical analyses

Genomic DNA was extracted from whole peripheral blood [26]. Genotyping of *A1CF* polymorphism (rs10821905) and *BAZ1B* polymorphism (rs1178977) was performed using a TaqMan assay (Applied Biosystems) employing a Lightcycler 480 (Roche Diagnostics) as reported in our previous study [27]. GWAS genotyping data were obtained from our previous study [10] which was performed using the Illumina HumanOmniExpress-12 v1.0 (Illumina) platform employing 945 clinically-ascertained cases and 1213 Japanese male controls.

For the calculations in the statistical analyses, we used R (version 4.0.1) [28] including a meta package [29] in a fixed effect model. The chi-squared test was used for the association and Hardy–Weinberg equilibrium analyses. A *P* value of < 0.05 was regarded as statistically significant.

## Results

Table 1 shows the genotyping results for rs10821905 of *A1CF* and rs1178977 of *BAZ1B* in 1,411 gout cases and 1,285 controls. The genotyping call rate for these SNPs exceeded 98%. These SNPs in the control group were in Hardy–Weinberg equilibrium (*P* > 0.05), which suggested no mistyping. Table 1 shows that both SNPs showed a significant association with gout.

**Table 1:** Association analysis between *A1CF* and *BAZ1B* and gout

Gene	SNP	Genotype^a^								Allele frequency mode	
		Case				Control				*P* value^b^	OR (95% CI)
		1/1	1/2	2/2	RAF	1/1	1/2	2/2	RAF		
*A1CF*	rs10821905	1252	150	3	0.0555	1168	106	2	0.0431	0.0366	1.30 (1.02–1.68)
*BAZ1B*	rs1178977	6	223	1174	0.916	14	240	1016	0.895	6.49 × 10^–3^	1.29 (1.07–1.55)

*SNP* single nucleotide polymorphism, *RAF* risk allele frequency, *OR* odds ratio, *CI* confidence interval

^a^The non-risk allele referred to as allele 1 and the risk allele as 2. Allele 1 is G and allele 2 is A in both rs10821905 and rs1178977

^b^*P* values were obtained by chi-squared tests

**Fig. 1:**
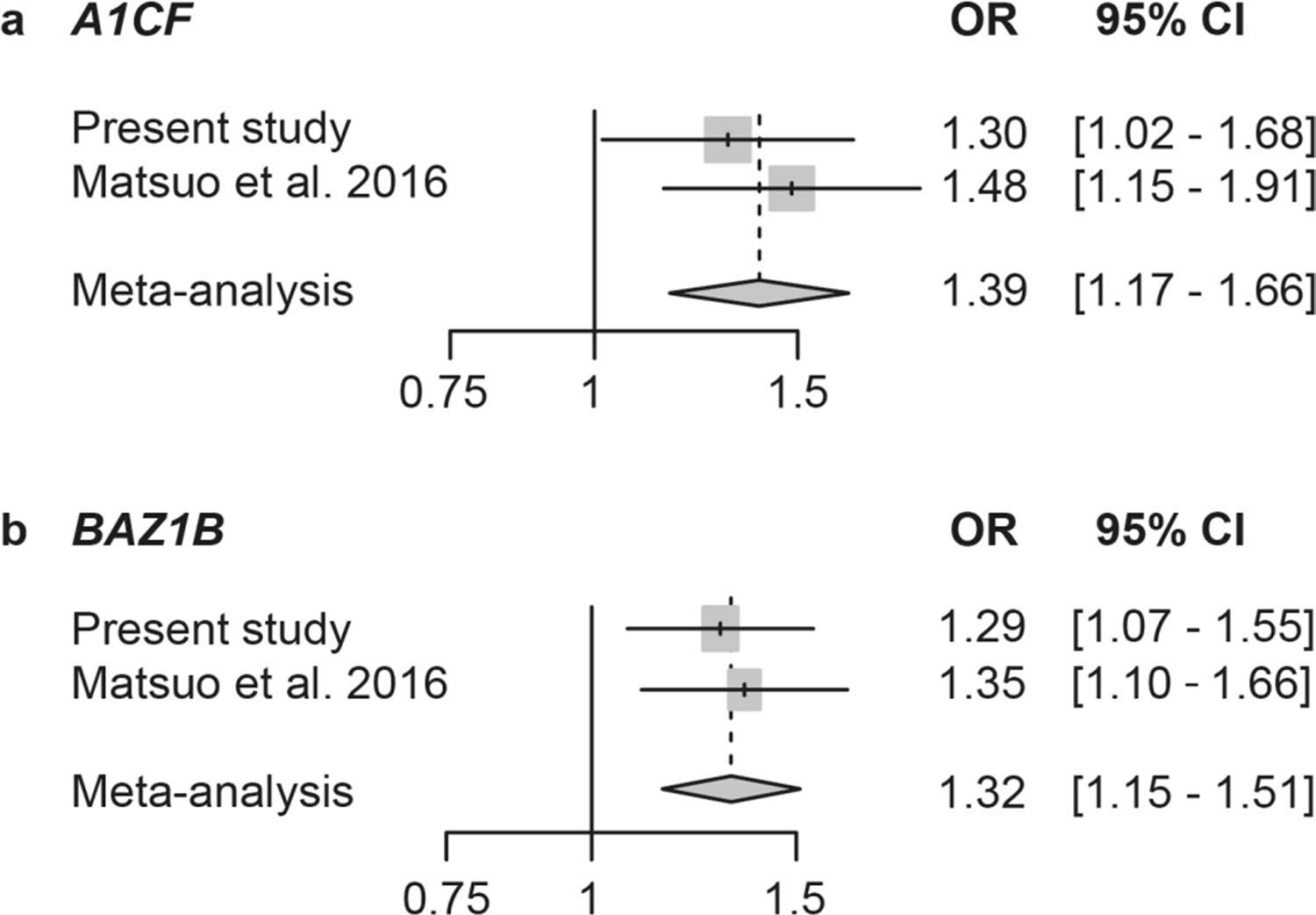
Meta-analysis of **a** rs10821905 of *A1CF* and **b** rs1178977 of *BAZ1B* for gout in the Japanese male population. The meta-analysis was conducted using the present study and our previous gout GWAS of Japanese male populations (Matsuo et al*.* [10]). Both SNPs showed a statistically significant association with gout (*P*_*meta*_ = 3.16 × 10^–4^ for rs10821905 of *A1CF*, *P*_*meta*_ = 7.28 × 10^–5^ for rs1178977 of *BAZ1B*). *OR* odds ratio, *CI* confidence interval

As shown in Fig. 1, the meta-analysis between the present study and previous gout GWAS [10] also showed both SNPs to have significant associations with gout (*P*_*meta*_ = 3.16 × 10^–4^, odds ratio [OR] with 95% confidential interval [CI]: 1.39 [1.16–1.66] for rs10821905 of *A1CF*, *P*_*meta*_ = 7.28 × 10^–5^, OR with 95% CI 1.32 [1.15–1.51] for rs1178977 of *BAZ1B*).

## Discussion

The present study showed, for the first time, an association between rs10821905 of *A1CF* as well as rs1178977 of *BAZ1B* and gout in a Japanese population.

Since it has been established that hyperuricemia is associated with dyslipidemia [30] such as hypertriglyceridemia [31], variants of *A1CF* may affect urate metabolism via the following ApoB-related mechanisms. *A1CF* (APOBEC1 complementation factor) encodes a complementation factor which forms a multi-component enzyme complex with APOBEC1 (apolipoprotein B mRNA editing enzyme catalytic subunit 1) to deaminate mammalian apolipoprotein B (ApoB) mRNA [32]. In other words, mRNA coding ApoB-100 is converted to mRNA coding ApoB-48 by this enzyme complex containing A1CF. Variants of *A1CF* may, therefore, affect urate metabolism through ApoB production and/or ApoB-related insulin resistance. Moreover, A1CF is reported to be expressed in the liver, kidney and intestine [33, 34], from where urate is also mainly produced and excreted in humans. Indeed, in addition to the present study, Dong et al*.* and Rasheed et al*.* have reported an association between gout and the *A1CF* variant in Han Chinese and in New Zealand European and Polynesian populations [35, 36]. Nevertheless, further studies need to be conducted to elucidate the precise pathophysiological background, when taking into account the fact that rs10821905 is located at about 2 kbp upstream of the *A1CF* gene. In other words, while the present study showed significance at rs10821905 of *A1CF*, it is possible that this is a mere marker and that the true risk gene is present close by.

This is the first report to identify an association between clinically-defined gout and *BAZ1B*. BAZ1B is possibly involved in urate metabolism due to transcriptional changes. *BAZ1B* (bromodomain adjacent to zinc finger domain 1B), also known as *WSTF* (Williams syndrome transcription factor), encodes a member of the bromodomain protein family. *BAZ1B* shows ubiquitous expression in humans and is mainly involved in the chromatin-dependent regulation of transcription, including chromatin assembly, RNA polymerase I and III gene regulation, vitamin D metabolism, and DNA repair [37]. Since Köttgen et al*.* [7] reported rs1178977 of *BAZ1B* to be in linkage disequilibrium (LD) with several SNPs of *MLXIPL*, it is probable that *BAZ1B* is a mere surrogate marker of *MLXIPL*, since MLXIPL/ChREBP is involved in the glucose-6-phosphate production, an upstream pathway of de novo urate production [38, 39]. However, because urate is an end metabolite of purine bodies including ATP and some nucleosides, it is also possible that changes in regulation of transcription by *BAZ1B* variants are associated with urate metabolism.

While deletion, including *BAZ1B*, is known to cause Williams syndrome [40], the mechanism between SUA or gout and *BAZ1B* variants remains to be elucidated. It is compatible in that the same SNP (rs1178977) is reported to have an association with triglyceride levels from a previous GWAS [41], when taking into account the association between urate and triglycerides [31].

We previously reported that ABCG2 (ATP-binding cassette subfamily G member 2) is a renal and intestinal urate exporter and that its dysfunctional variants have a significant and strong effect on susceptibility to gout/hyperuricemia [8, 42, 43]. We, therefore, recalculated the results shown in Table 1 with and without these variants. As shown in Supplementary Table S1, the *A1CF* variant still showed a significant association with gout in the presence of the dysfunctional variants of ABCG2 but was no longer significant without those variants, while the *BAZ1B* variant remained significant both with and without ABCG2 dysfunction. *A1CF* might, therefore, have synergistic effects on susceptibility to gout with dysfunctional variants of *ABCG2*, for which further analyses are necessary.

As mentioned in the Introduction, both *A1CF* and *BAZ1B* were first detected in the GWAS of SUA from a European population with genome-wide significance [*P* = 7.40 × 10^–17^, beta = 0.057, and *P* = 1.20 × 10^–12^, beta = 0.0247 (unit: mg/dl)]. These SNPs also showed nominal significance with gout [*P* = 0.026, OR = 1.09, and *P* = 6.70 × 10^–4^, OR = 1.14, respectively] [7]. Our previous GWAS of SUA from a Japanese population also showed [44] a nominally significant association (*P* = 1.79 × 10^–3^, beta = 0.029, and *P* = 2.35 × 10^–7^, beta = 0.033). The present result and the previous reports suggest there to be a shared physiological or pathophysiological background between Japanese and European populations for both SUA increase and gout susceptibility.

Our findings should not only assist the elucidation of the pathophysiology of gout and hyperuricemia, but also suggests new molecular targets.

## Supplementary Information

Below is the link to the electronic supplementary material.

Supplementary file1 (PDF 85 KB)
