# Supplementary material for: Both variants of A1CF and BAZ1B genes are associated with gout susceptibility: a replication study and meta-analysis in a Japanese population
Source: Hum Cell. 2021 Jan 31;34(2):293–9. doi: 10.1007/s13577-021-00485-4 (PMC7900071; doi:10.1007/s13577-021-00485-4)
Supplement: Supplementary file 1 — Supplementary file1 (PDF 85 KB) [file 13577_2021_485_MOESM1_ESM.pdf]

Supplementary Information

Both variants of *A1CF* and *BAZ1B* genes are associated with gout susceptibility: A replication study and meta-analysis in a Japanese population

Makoto Kawaguchi, Akiyoshi Nakayama, *et al.*
Correspondence to Hirotaka Matsuo (email: hmatsuo.ndmc@gmail.com)

Supplementary Table S1 Association analysis between *A1CF* and *BAZ1B* and gout with and without dysfunctional variants of "gout gene" *ABCG2*.

Gene	Dysfunctional variants of *ABCG2*[a]	Genotype[b]								Allele frequency mode	
		Case				Control				*p* value[c]	OR (95% CI)
		1/1	1/2	2/2	RAF	1/1	1/2	2/2	RAF		
A1CF	(+)	925	114	3	0.0576	581	47	1	0.0390	0.0172	1.51 (1.07 – 2.12)
	(-)	327	36	0	0.0496	587	59	1	0.0471	0.805	1.05 (0.69 – 1.61)
BAZ1B	(+)	4	173	862	0.913	8	121	496	0.890	0.0325	1.29 (1.02 – 1.63)
	(-)	2	50	312	0.926	6	119	520	0.898	0.0407	1.41 (1.01 – 1.96)

RAF risk allele frequency; *OR*, odds ratio; *CI*, confidence interval.

[a]The two commonest dysfunctional variants of *ABCG2*, Q126X (rs72552713) and Q141K (rs2231142), were investigated (ref. 8 in the main text).

[b]The non-risk allele referred to as allele 1 and the risk allele as 2. Allele 1 is G and allele 2 is A in both rs10821905 of *A1CF* and rs1178977 of *BAZ1B*.

[c]*P*-values were obtained by chi-squared tests.
